# Molecular Epidemiology and Genetic Evolution Analysis of Porcine Reproductive and Respiratory Syndrome Virus in Southern Xinjiang, China, from 2023 to 2025

**DOI:** 10.3390/vetsci12090874

**Published:** 2025-09-09

**Authors:** Shuhua Liu, Mengzhe Hou, Xin Chen, Baihe Ma, Zhen Zhang, Meiliang Guo, Yunlai Chen, Lianrui Li

**Affiliations:** 1 College of Animal Science and Technology, Tarim University, Alar 843300, China; 10757232113@stumail.taru.edu.cn (S.L.); 10757232139@stumail.taru.edu.cn (M.H.); 10757231074@stumail.taru.edu.cn (X.C.); 10757242145@stumail.taru.edu.cn (B.M.); 10757242138@stumail.taru.edu.cn (Z.Z.); 10757242124@stumail.taru.edu.cn (M.G.); 2221221238@stumail.taru.edu.cn (Y.C.); 2 Key Laboratory of Tarim Animal Husbandry Science and Technology, Xinjiang Production & Construction Corps, Alar 843300, China; 3 Engineering Laboratory of Tarim Animal Diseases Diagnosis and Control, Xinjiang Production & Construction Corps, Alar 843300, China

**Keywords:** PRRSV-1/2, xinjiang, China, NADC30-like, ORF5 gene, next-generation sequencing

## Abstract

Porcine reproductive and respiratory syndrome virus (PRRSV), a major pathogen causing substantial economic losses in the global swine industry, was studied in southern Xinjiang to characterize its local epidemic features. Based on 632 clinical samples collected from 13 pig farms between 2023 and 2025, quantitative RT-PCR detection showed an overall positivity rate of 18.35% (116/632), with PRRSV-2 single infection accounting for 97.41% (113/116), PRRSV-1 single infection for 1.72% (2/116), and co-infection for 0.86% (1/116). Among 38 ORF5 sequences obtained from positive samples, Sublineage 1.8 (NADC30-like) was dominant, comprising 97.14% of successfully sequenced strains. Molecular analysis revealed that PRRSV-1 isolates carried six amino acid mutations including A129V (consistent with the Chinese strain CN/FJFQ-1/2023), while PRRSV-2 strains exhibited key variations such as the neutralization escape mutation Q13R, virulence-associated site K151R, and an anomalous vaccine marker A137. Furthermore, a recombinant strain (XJLETUQ2025-7) between NADC30 and VR-2332 was identified with breakpoints in NSP2 and NSP10. Serological surveillance of 2043 vaccinated pigs showed an overall antibody positive rate of 83.0% (1696/2043), with increasing annual rates from 72.3% (2023) and 75.4% (2024) to 91.3% (2025). In conclusion, the PRRSV epidemic in southern Xinjiang is primarily driven by NADC30-like strains of PRRSV-2, with recombination events and GP5 epitope variations posing challenges to disease control. These findings enhance the epidemiological understanding of PRRSV in the region and provide valuable insights for vaccine development and prevention strategies.

## Simple Summary

Elucidating the Epidemiology of PRRSV in Southern Xinjiang is critical for global pandemic prevention and control. This study was conducted based on pathogen detection (RT-qPCR/sequencing) of 632 clinical samples collected from 2023 to 2025, along with antibody monitoring (ELISA) of 2043 serum samples from PRRSV-vaccinated pigs. Results: The dominant strain was PRRSV-2 NADC30-like (Sublineage 1.8), accounting for 97.14% of successfully sequenced samples; Evolutionary markers: PRRSV-1 carried the A129V (GP5) mutation, consistent with Chinese isolates; PRRSV-2 exhibited a Q13R neutralization escape mutation and an anomalous A137 vaccine marker; Recombination mechanism: Identification of the XJLETUQ2025-7 recombinant (NADC30/VR-2332, breakpoints: NSP2/NSP10); Immunization outcome: Antibody protection remained at a relatively high level. Conclusions: The findings reveal that the PRRSV epidemic in southern Xinjiang is dominated by NADC30-like recombinant strains, with GP5 epitope variations and vaccine marker shifts providing precise targets for prevention and control strategies.

## 1. Introduction

Porcine Reproductive and Respiratory Syndrome (PRRS) is a severe viral disease caused by the Porcine Reproductive and Respiratory Syndrome Virus (PRRSV). It has long been responsible for significant economic losses in the global swine industry. The disease is clinically characterized by reproductive failure in sows—such as abortions, stillbirths, and mummified fetuses—and respiratory disorders in piglets [1]. Since its initial identification, PRRS has been circulating worldwide for over 30 years and remains a major threat to swine health [2]. The disease first emerged in the late 1980s in the United States and Europe, where it was initially referred to as “blue-ear pig disease”, “porcine endemic abortion and respiratory syndrome” or “mystery swine disease”. PRRS is highly contagious and can lead to high mortality rates [3,4]. Through epidemiological investigations and validation via Koch’s postulates, scientists confirmed that the viral strains isolated from the initial outbreaks were PRRSV and established it as the causative agent of PRRS [5].

PRRSV belongs to the order Nidovirales, family Arteriviridae, and genus Arterivirus. Its genome is a non-segmented, single-stranded positive-sense RNA, approximately 15.4 kb in length, containing at least 12 open reading frames (ORFs) that encode eight structural proteins and at least 16 non-structural proteins [6]. From the 5′ end to the 3′ end, the genome is composed of ORF1a, ORF1b, ORF2a, ORF2b, ORF3, ORF4, ORF5a, ORF5, ORF6, and ORF7. Among them, ORF1a and ORF1b, located at the 5′ end, account for approximately 80% of the total genome length and are responsible for encoding replication enzyme proteins with RNA polymerase activity [7]. ORF5 encodes the envelope glycoprotein (GP5) of the virus, ORF6 encodes the membrane protein (M), and ORF7 encodes the nucleocapsid protein (N). GP5 and M are the main targets for antigenic epitope research [8,9,10]. Notably, ORF5 is commonly used for PRRSV genotyping, genetic variation analysis, and evolutionary studies [11].

PRRSV can be divided into two main genotypes: the European genotype (genotype 1), with Lelystad virus (LV) as the prototype strain; and the North American genotype (genotype 2), with VR-2332 as the prototype strain. Based on the global PRRSV classification system and ORF5 sequence analysis, PRRSV-2 can be further divided into nine different lineages (Lineage 1–9), each containing multiple sublineages [12,13,14]. Since PRRSV was first discovered in China in 1996, PRRSV-2 strains have dominated Chinese pig populations [15,16,17]. In 2006, the emergence of Highly Pathogenic PRRSV (HP-PRRSV) triggered a large-scale epidemic of atypical PRRS in China, resulting in pig mortality rates as high as 20–80%. NADC30-like PRRSV was reported in China in 2013 and is believed to have originated from North American imports and adapted locally, spreading rapidly in China in recent years [18,19]. Although PRRSV-1 strains have also been detected in China [20,21,22], PRRSV-2 strains currently dominate Chinese pig populations, mainly including Lineages 1, 3, 5, and 8, with recombination between Lineage 1 and Lineage 8 being particularly common [23,24]. The continuous genetic variation and recombination of PRRSV lead to the emergence and spread of new strains, posing significant challenges and complexities for clinical prevention and control efforts.

However, research reports on the prevalence and genetic diversity of PRRSV in Xinjiang, China, are relatively scarce, and they are primarily focused on the northern part of Xinjiang. The prevalence of PRRSV in the southern part of Xinjiang in recent years remains unclear. To fill this knowledge gap and clarify the characteristics and genetic diversity of PRRSV epidemic strains in the southern part of Xinjiang, this study collected diseased pig samples from the southern part of Xinjiang between 2023 and 2025. Firstly, fluorescent quantitative PCR (fluorescent PCR) was used for PRRSV detection. Subsequently, reverse transcription polymerase chain reaction (RT-PCR) was performed on positive samples to amplify the ORF5 gene, followed by nucleotide sequencing and genetic evolution analysis. The obtained ORF5 nucleotide sequence and its deduced GP5 protein amino acid sequence were compared with PRRSV reference strains from the United States, the Netherlands, and China. In addition, this study utilized next-generation sequencing technology (Next-generation sequencing, NGS) to directly sequence clinical samples to obtain whole-genome sequences, ensuring the originality of the data, and conducted whole-genome sequence analysis. Finally, enzyme-linked immunosorbent assay (ELISA) was used to monitor the PRRSV antibody levels in serum samples collected between 2023 and 2025.

## 2. Materials and Methods

### 2.1. Sample Collection

This study was conducted from 2023 to 2025 in the southern region of Xinjiang, involving 13 large-scale pig farms as research subjects. All participating farms voluntarily took part and signed informed consent forms. A total of 632 clinical samples were collected for the detection of Porcine Reproductive and Respiratory Syndrome Virus (PRRSV), covering all 13 cooperating pig farms. The number of samples from each farm varied based on clinical availability, ranging from 25 to 75. Sample types included the following: boar semen, placental tissues and oral fluids from aborting sows, tongue tip fluids from aborted piglets, fluids from castration of nursery piglets, as well as tissues (such as lungs and lymph nodes) and whole blood from deceased piglets and pigs suspected of PRRSV infection. Additionally, 2043 serum samples from PRRSV-vaccinated pigs were collected during the same period for antibody level monitoring. All samples were obtained following the principle of clinical convenience during routine veterinary procedures on the farms. After collection, samples were immediately placed in −20 °C ice boxes for transport to the laboratory. Clinical tissue samples were stored in −80 °C ultra-low temperature freezers, while immune serum samples were stored at 4 °C. All virus-related procedures, including nucleic acid extraction, were conducted in a Biosafety Level 2 (BSL-2) laboratory, strictly adhering to biosafety protocols to prevent pathogen dissemination and cross-contamination.

### 2.2. Fluorescence Quantitative PCR Detection for PRRSV Clinical Samples

Viral RNA was extracted from the samples using the *EasyPure*^®^ Viral DNA/RNA Kit (TransGen Biotech, Beijing, China) according to the manufacturer’s instructions. Subsequently, the extracted viral RNA was detected and analyzed using a commercial Porcine Reproductive and Respiratory Syndrome Virus (North American Strain/European Strain) Duplex Real-Time RT-PCR Detection Kit (Hunan Guanmu, Hunan, China) on the QuantGene 9600 Real-Time PCR System (Hangzhou Bioer, Zhejiang, China).

### 2.3. Amplification and Sequencing of ORF5 Gene from PRRSV-Positive Nucleic Acid

The nucleic acid from samples identified as positive in the previous detection step was reverse transcribed into cDNA using the *EasyScript*^®^ One-Step gDNA Removal and cDNA Synthesis SuperMix (TransGen Biotech, Beijing, China). To analyze the PRRSV ORF5 gene sequence, specific primers were designed for PRRSV-1 and PRRSV-2: primers for PRRSV-1 were designed according to reference [25], while primers for PRRSV-2 were designed based on reference sequences from GenBank. Both designed primer pairs amplified fragments containing the complete ORF5 gene (Table 1) using a high-fidelity DNA polymerase, yielding products of 702 bp for PRRSV-1 and 963 bp for PRRSV-2. Amplification was performed in a Bio-Rad T100 Thermal Cycler (Bio-Rad, USA). The 50 μL PCR reaction mixture contained 25 μL of 2× *TransStart*^®^ *FastPfu* Fly PCR SuperMix (TransGen Biotech, Beijing, China), 1 μL each of forward and reverse primers, 2 μL of cDNA template, and 21 μL of ddH_2_O. The PCR parameters for PRRSV-1 were as follows: initial denaturation at 98 °C for 1 min; 35 cycles of denaturation at 98 °C for 10 s, annealing at 52 °C for 5 s, and extension at 72 °C for 4 s; followed by a final extension at 72 °C for 1 min. The PCR parameters for PRRSV-2 were as follows: initial denaturation at 98 °C for 1 min; 35 cycles of denaturation at 98 °C for 10 s, annealing at 57 °C for 5 s, and extension at 72 °C for 5 s; followed by a final extension at 72 °C for 1 min. Positive PCR products were purified using the TIANgel Midi Purification Kit (TIANGEN, Beijing, China), and subsequently cloned into the *pEASY*^®^-Blunt Zero Cloning Vector using the *pEASY*^®^-Blunt Zero Cloning Kit (TransGen Biotech, Beijing, China). Positive recombinant plasmids were submitted to Sangon Biotech (Shanghai, China) for sequencing, with all sequencing reactions performed in triplicate.

### 2.4. High-Throughput Sequencing (NGS) of the PRRSV Whole Genome

Viral nucleic acid extracts were selected from samples originating from pig farms exhibiting higher PRRSV-associated mortality in epidemiological investigations. Specifically, samples were chosen based on a positive result in the PRRSV fluorescence quantitative RT-PCR assay with a cycle threshold (Ct) value < 25. These selected nucleic acid samples were subsequently commissioned to Sangon Biotech (Shanghai, China) for the determination of the complete PRRSV genome sequence using next-generation sequencing (NGS) technology. Following the acquisition of the full-length sequences, bioinformatics analysis was performed.

### 2.5. Sequence Alignment and Phylogenetic Analysis

Representative PRRSV strain sequences (ORF5 gene and complete genomes) were retrieved from the GenBank database (Table 2). Using MEGA 12 software, the PRRSV strain sequences obtained in this study (both ORF5 gene and whole genomes) were aligned with the aforementioned reference sequences through multiple sequence alignment. Phylogenetic trees were then constructed employing the Neighbor-Joining (NJ) method. Nucleotide sequence homology analysis of the ORF5 genes was performed using the MegAlign module in DNASTAR 7.1 software, and the deduced amino acid sequences were also aligned. To detect potential genetic recombination events in the PRRSV strains, reference strains from NCBI (ATCC-VR2332, NADC30, CH-1a, GM2, JXA1, IA/2014/NADC34) and the strain sequences from this study were analyzed using RDP 4 software for recombination signal prediction. Strains identified by RDP4 as potential recombinants were further analyzed with SimPlot 3.5 software to verify the locations of recombination breakpoints.

### 2.6. PRRSV Antibody Monitoring

Serum samples were collected and screened for PRRSV antibodies using a commercial Porcine Reproductive and Respiratory Syndrome Virus (PRRSV) ELISA antibody detection kit (Jinnuo Diagnostics, Beijing, China). Strictly adhering to the manufacturer’s instructions, results were read and analyzed using an ELISA reader at 450 nm absorbance. Results were interpreted as positive or negative based on the S/P ratio, calculated using the following formula: S/P = (Sample OD450 nm value—Mean NC OD450 nm)**/**(Mean PC OD450 nm—Mean NC OD450 nm). Samples with an S/P ratio ≥ 0.4 were considered positive, while those with an S/P ratio < 0.4 were considered negative.

## 3. Results

### 3.1. Virus Detection and Genetic Evolutionary Analysis of ORF5 Gene

**Figure 1:**
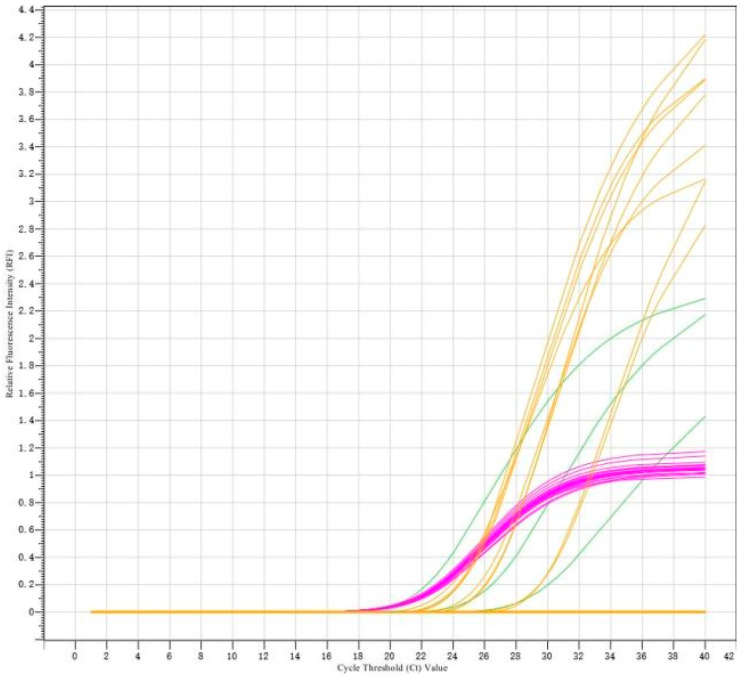
Amplification curves of the duplex real-time RT-PCR for simultaneous detection of PRRSV-1 and PRRSV-2. Curves represent fluorescence signals from orange: PRRSV-2 (North American genotype), positive control (PC); green: PRRSV-1 (European genotype), PC; and purple: internal control (IC).

**Figure 2:**
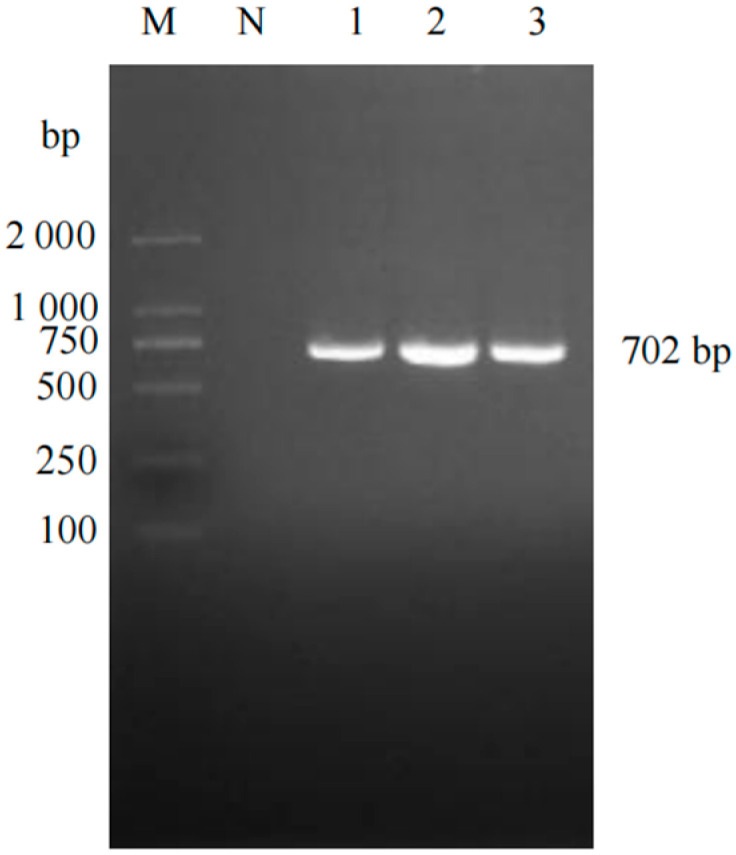
PCR results of PRRSV-1-positive samples. M: DL2000 Marker; NC: Negative control; Lanes 1–3: Positive samples.

**Figure 3:**
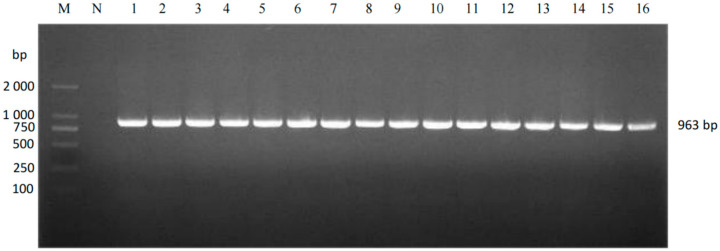
PCR results of selected PRRSV-2-positive samples. M: DL2000 Marker; NC: Negative control; Lanes 1–16: Positive samples.

**Figure 4:**
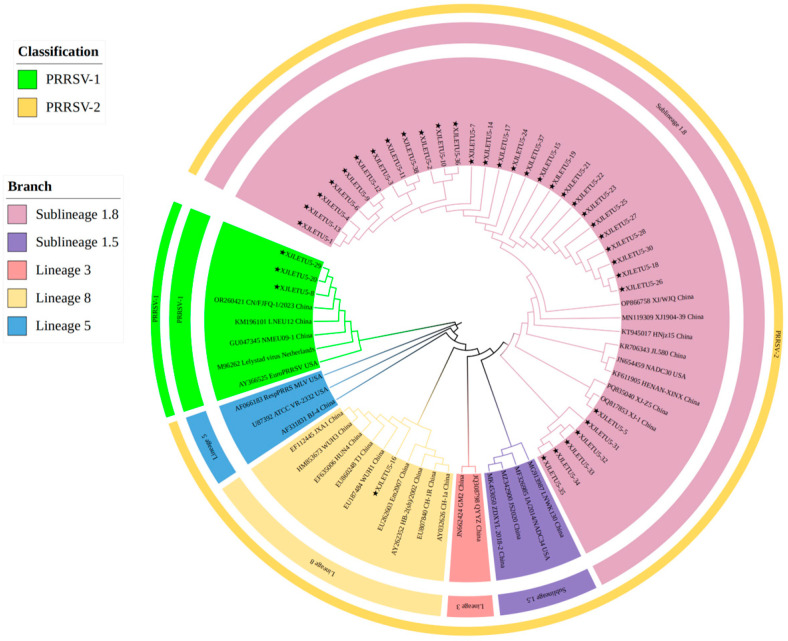
Phylogenetic tree constructed based on the nucleotide sequences of the Open Reading Frame 5 (ORF5) gene from PRRSV strains and reference strains. Phylogenetic analysis was performed using the Neighbor-Joining (NJ) method in MEGA 12 software, with 1000 bootstrap replicates to assess node support values. The pentagram symbols represent the PRRSV strains obtained in this study, while the others are reference strains retrieved from the GenBank database. The final tree was visually optimized using the iTOL online tool to improve readability and identification efficiency.

A total of 632 clinical samples collected from southern Xinjiang between 2023 and 2025 were tested using a commercial duplex real-time RT-PCR kit for Porcine Reproductive and Respiratory Syndrome Virus (North American/European strains). The results revealed 116 samples were PRRSV nucleic acid-positive (overall positivity rate: 18.35%, 116/632) (Figure 1). Subtyping of positive samples indicated that single PRRSV-2 infection accounted for 97.41% (113/116), single PRRSV-1 infection accounted for 1.72% (2/116), and mixed infection accounted for 0.86% (1/116). To characterize the genetic features of the prevalent strains, 38 full-length ORF5 gene sequences were successfully obtained from the positive samples (PRRSV-1: 3; PRRSV-2: 35) (Figure 2 and Figure 3). Neighbor-Joining phylogenetic analysis (Figure 4) revealed that among the PRRSV-2 strains, 34 isolates (97.14%) were assigned to Sublineage 1.8, while one isolate (2.86%) belonged to Lineage 8. All three PRRSV-1 strains clustered within the European classical branch represented by the Lelystad virus, indicating that NADC30-like strains are predominant in southern Xinjiang with a higher clinical detection rate.

### 3.2. Homology Analysis of PRRSV ORF5 Gene

**Figure 5:**
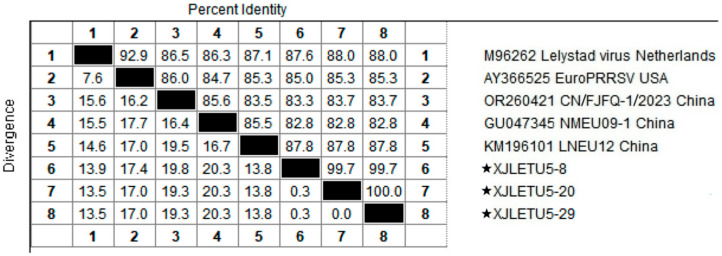
Homology analysis of PRRSV-1 nucleotide sequences based on the ORF5 gene (generated using DNASTAR 7.1 MegAlign). The pentagram symbols represent the PRRSV strains obtained in this study, while the others denote reference strains retrieved from the GenBank database.

**Figure 6:**
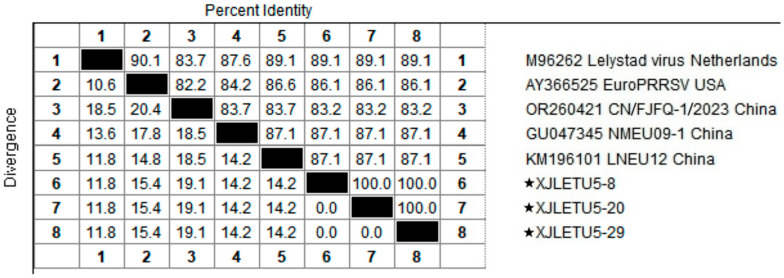
Homology analysis of PRRSV-1 amino acid sequences based on the ORF5 gene (generated using DNASTAR 7.1 MegAlign). The pentagram symbols represent the PRRSV strains obtained in this study, while the others denote reference strains retrieved from the GenBank database.

**Figure 7:**
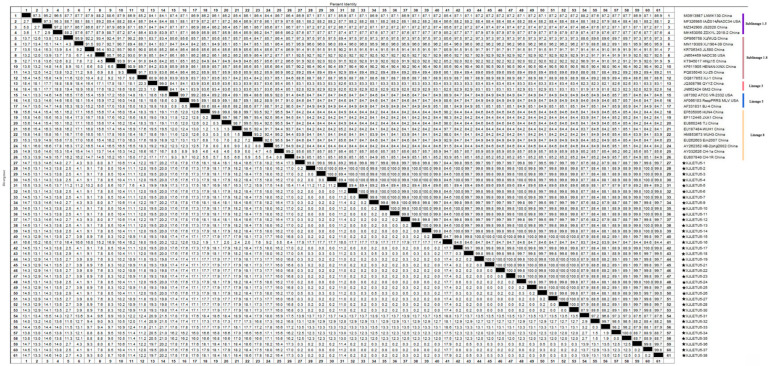
Homology analysis of PRRSV-2 nucleotide sequences based on the ORF5 gene (generated using DNASTAR 7.1 MegAlign). The pentagram symbols denote the PRRSV strains obtained in this study, while the others represent reference strains retrieved from the GenBank database.

**Figure 8:**
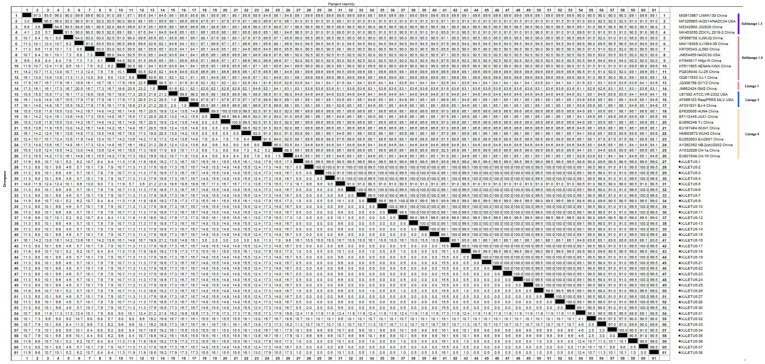
Homology analysis of PRRSV-2 amino acid sequences based on the ORF5 gene (generated using DNASTAR 7.1 MegAlign). The pentagram symbols represent the PRRSV strains obtained in this study, while the others denote reference strains retrieved from the GenBank database.

Homology analysis of the ORF5 gene of the obtained PRRSV strains with reference sequences from both domestic and international sources revealed that the nucleotide homology among the three PRRSV-1 strains ranged from 99.7% to 100%, with 100% amino acid homology. However, their nucleotide homology with representative strains such as Lelystad virus, EuroPRRSV, CN/FJFQ-1/2023, NMEU09-1, and LNEU12 was only 82.8% to 88.0%, with amino acid homology ranging from 82.2% to 89.1% (Figure 5 and Figure 6). Among the 35 PRRSV-2 strains, the nucleotide homology ranged from 83.7% to 100%, with amino acid homology from 84.6% to 100%. When compared with 26 representative strains, including ATCC-VR2332, NADC30, CH-1a, GM2, JXA1, and IA/2014/NADC34, the nucleotide homology ranged from 81.8% to 98.3%, with amino acid homology from 81.1% to 97.5% (Figure 7 and Figure 8). These results indicate a high degree of genetic diversity in the PRRSV ORF5 gene in southern Xinjiang.

### 3.3. Mutation Analysis of ORF5 Gene-Encoded Protein

**Figure 9:**
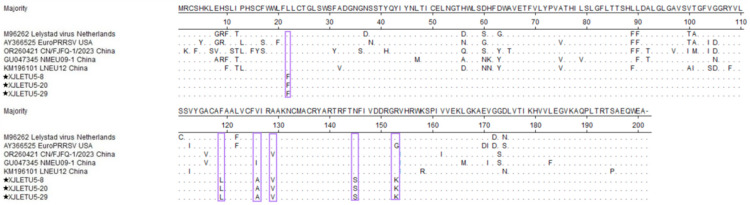
Alignment of GP5 protein sequences of PRRSV-1 strains (generated using DNASTAR 7.1 MegAlign). The pentagram symbols represent the PRRSV strains obtained in this study, while the others denote reference strains retrieved from the GenBank database.

The GP5 protein sequence alignment of the PRRSV-1 strain (ORF5 encoding 201 aa) showed (Figure 9) that there were six mutation sites in the three strains in Xinjiang, mainly manifested as L22F, F119L, V126A, A129V, N145S, and R153K. Among them, 129 mutation sites were consistent with the representative strain CN/FJFQ-1/2023 in China, suggesting that the Xinjiang strain may have originated from a local evolutionary branch in China.

**Figure 10:**
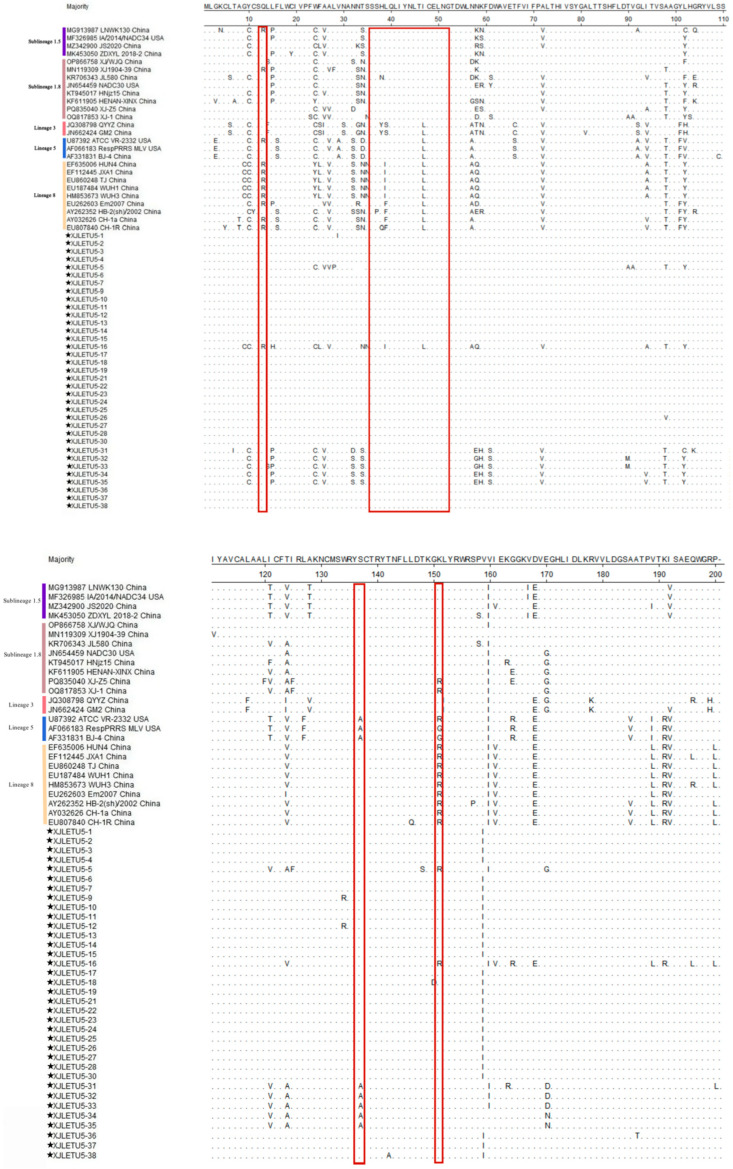
Alignment of GP5 protein sequences of PRRSV-2 strains (generated using DNASTAR 7.1 MegAlign). The pentagram symbols represent the PRRSV strains obtained in this study, while the others denote reference strains retrieved from the GenBank database.

Sequence alignment of the GP5 protein of PRRSV-2 strains (ORF5 encoding 200 aa) revealed (Figure 10) that multiple mutation sites were present in the GP5 protein of 35 strains from southern Xinjiang. In this experiment, one strain exhibited a Q13R mutation, belonging to Lineage 8 (CH-1a and JXA1-like). Two strains showed a K151R mutation, one belonging to Sublineage 1.8 (NADC30-like) and the other to Lineage 8 (CH-1a and JXA1-like). When the 13th amino acid of GP5 mutates from glutamine to arginine or the 151st amino acid mutates from lysine to arginine, the strain exhibits virulent characteristics [26,27,28,29]. Mutations in the neutralizing epitope region encoded by ORF5, spanning amino acids 36–52, may lead to the virus evading the neutralizing effect induced by vaccine immunity, thereby reducing the protective efficacy of vaccine immunization. In this study, one strain exhibited a mutation in the neutralizing epitope region, belonging to Lineage 8 (CH-1a and JXA1-like). The 137th amino acid (A137) of GP5 is unique to vaccine strains and is often considered a site for distinguishing wild-type strains from vaccine strains [27,29,30,31]. In this study, five NADC30-like isolated strains were found to exhibit A137, which may have resulted from recombination or mutation.

### 3.4. High-Throughput Sequencing (NGS) and Whole-Genome Sequence Analysis

One European strain and two American strains were subjected to high-throughput sequencing. Each sample underwent two technical replicates. A third-party company utilized an Illumina high-throughput sequencer (Illumina, USA)to complete 2 × 150 bp dual-end sequencing, followed by assembly. The results indicated that samples 5 and 6 could be de novo assembled into the full-length sequence of PRRSV. After error correction, a consensus sequence would be obtained. The remaining samples failed to assemble into complete sequences.

#### 3.4.1. Whole-Genome Genetic Evolution and Homology Analysis

**Figure 11:**
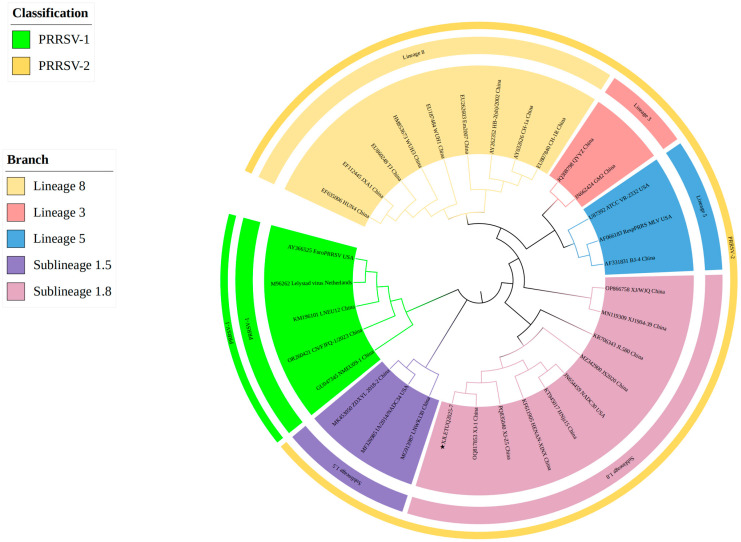
Phylogenetic tree constructed based on the complete genome nucleotide sequences of PRRSV strains and reference strains. Phylogenetic analysis was performed using the Neighbor-Joining (NJ) method in MEGA 12 software with 1000 bootstrap replicates to assess node support values. The pentagram symbols represent the PRRSV strains obtained in this study, while the others denote reference strains retrieved from the GenBank database. The final tree was visually optimized using the iTOL online tool to improve readability and interpretation efficiency.

**Figure 12:**
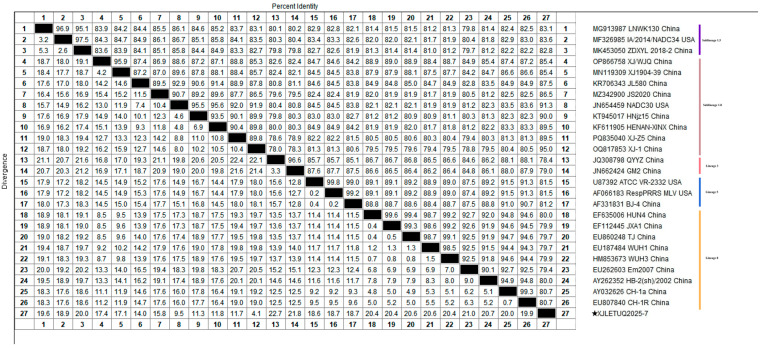
Nucleotide homology analysis of PRRSV-2 based on complete genome sequences (generated using DNASTAR 7.1 MegAlign). The pentagram symbols represent the PRRSV strains obtained in this study, while the others denote reference strains retrieved from the GenBank database.

**Figure 13:**
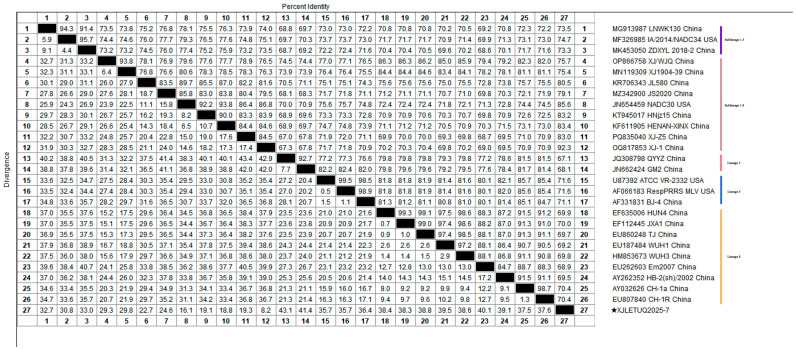
Amino acid homology analysis of PRRSV-2 based on complete genome sequences (generated using DNASTAR 7.1 MegAlign). The pentagram symbols represent the PRRSV strains obtained in this study, while the others denote reference strains retrieved from the GenBank database.

A full-length genomic sequence (15,044 bp) of PRRSV was successfully assembled from samples collected in southern Xinjiang, designated as XJLETUQ2025-7, with an average sequencing depth > 100× and genome coverage > 99.99%. Phylogenetic analysis (Figure 11) revealed that this strain belongs to PRRSV-2 Sublineage 1.8 (NADC30-like). It exhibited nucleotide sequence identities of 78.4–95% and amino acid sequence identities of 67.1–92.3% when compared with 26 reference strains of PRRSV-2. The highest homology was observed with the locally isolated Xinjiang strain XJ-1 (NADC30-like), with nucleotide and amino acid identities reaching 95% and 92.3%, respectively. Together, they form a distinct regional evolutionary cluster (Figure 12 and Figure 13).

#### 3.4.2. Recombinant Analysis

**Figure 14:**
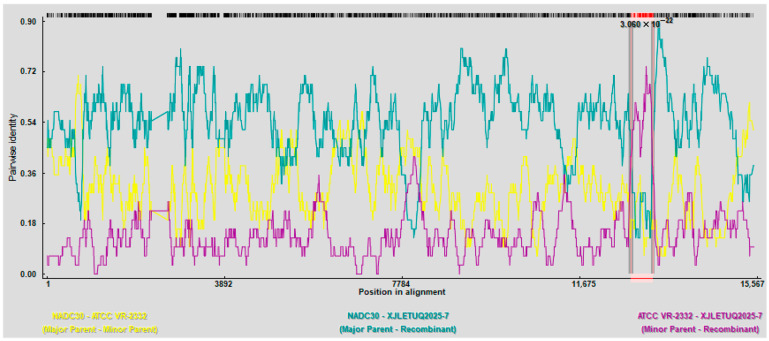
Recombination analysis of strain XJLEYUQ2025-7 (using RDP4 software).

**Figure 15:**
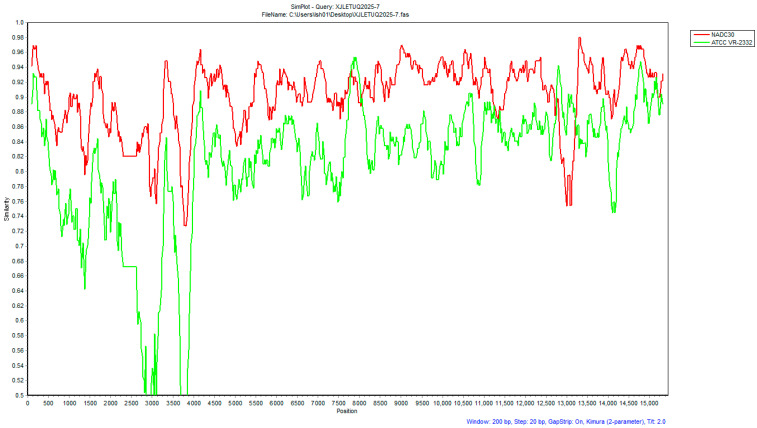
Recombination breakpoint analysis of strain XJLEYUQ2025-7 (conducted using SimPlot 3.5 software).

Recombination signals in XJLEYUQ2025-7 were scanned using the RDP4 software (employing seven algorithms: RDP, GeneConv, BootScan, MaxChi, Chimera, SiScan, and 3Seq). All methods consistently confirmed major parent strain: NADC30; minor parent strain: ATCC VR-2332 (Figure 14). Recombination breakpoints were further verified using SimPlot 3.5 software, which identified two recombinant regions: 7781–8021 nt (NSP2) and 12,741–13,201 nt (NSP10) (Figure 15).

### 3.5. Antibody Level Monitoring

**Figure 16:**
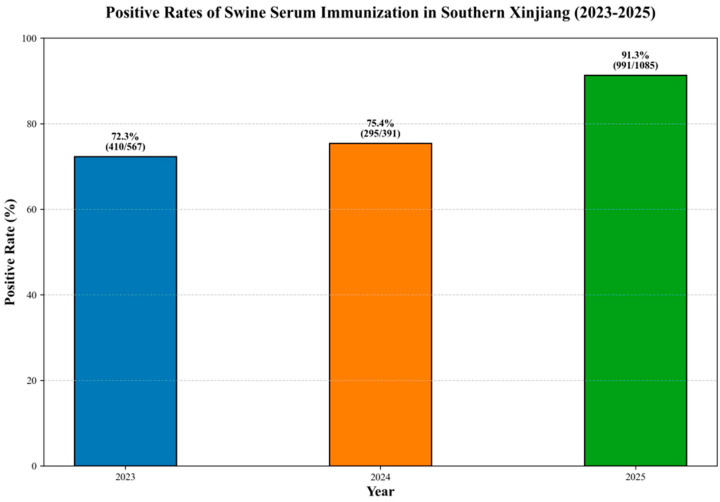
Antibody level monitoring in southern Xinjiang from 2023 to 2025.

A PRRSV-specific antibody detection assay was performed on 2043 serum samples collected from vaccinated swine across 13 farms in southern Xinjiang between 2023 and 2025. The results revealed an overall antibody positivity rate of 83.0% (1696/2043). Dynamic annual analysis showed that the positivity rate was 72.3% (410/567) in 2023, 75.4% (295/391) in 2024, and increased to 91.3% (991/1085) in 2025 (Figure 16), suggesting a high level of immune protection provided by the PRRSV vaccine in the southern Xinjiang region.

## 4. Discussion

Xinjiang, situated in northwestern China and sharing borders with multiple countries, has a pig industry that serves as a pivotal sector in local animal husbandry, holding strategic significance for the rural economy and farmers’ income. Since the late 1980s, Porcine Reproductive and Respiratory Syndrome Virus (PRRSV) has caused substantial economic losses worldwide due to its high transmissibility and mutation capacity. The prevalence of PRRSV in China is complex, with PRRSV-2 comprising multiple branches such as Lineage 1, 3, 5, and 8, while PRRSV-1 has also been sporadically reported [32]. Following the initial report of NADC30-like strains in China in 2012, this variant rapidly disseminated [33]. Junhui Li et al. [34] reported that the PRRSV positivity rate in pig farms in northern Xinjiang from 2020 to 2022 reached 53.6%, with the dominant strain being a recombinant NADC30-like variant, consistent with the findings from southern Xinjiang in this study.

PRRSV GP5 is associated with viral replication, virion assembly, and neutralizing antibody production. Consequently, deletions, insertions, or mutations in the GP5 amino acid sequence can alter viral replication and vaccine efficacy [35,36]. In this study, alignment of GP5 protein amino acid sequences using DNASTAR MegAlign revealed that the GP5 protein encoded by PRRSV-1 ORF5 at position 129 is identical to that of the Chinese Fujian strain CN/FJFQ-1/2023, suggesting that the Xinjiang strain may have originated from a domestic evolutionary branch. Analysis of key virulence sites showed that one Lineage 8 strain harbored a Q13R mutation, and two strains (belonging to Sublineage 1.8 and Lineage 8, respectively) carried a K151R mutation, both of which are associated with enhanced virulence [26,27,28,29]. Additionally, one Lineage 8 strain exhibited a mutation in the neutralizing epitope region (36–52 aa), which may mediate immune escape. Notably, five NADC30-like isolates carried the vaccine strain characteristic site A137, indicating potential recombination or mutation events [27,29,30,31]. In recent years, reports of complex recombination events involving NADC30-like strains have significantly increased [18,37,38]. Given that ORF5 accounts for only about 4% of the viral genome [39,40], whole-genome sequencing (WGS) is crucial for elucidating viral evolution. This study obtained the full genome of a recombinant between NADC30-like and VR-2332, which, combined with data from northern Xinjiang [34], demonstrates that recombination is an important mechanism in the evolution of PRRSV in Xinjiang. Furthermore, PRRSV-1 was detected in southern Xinjiang, including two cases of single infection and one case of mixed infection, indicating that this subtype has established local transmission chains. Continuous monitoring is necessary to prevent new risks arising from recombination with PRRSV-2. However, whole-genome sequencing of the PRRSV-1-positive clinical samples did not yield complete genome sequences. To further investigate the characteristics of the virus, we attempted to isolate the virus by inoculating PRRSV-1-positive materials into PAM cells and Marc-145 cells, but these efforts were also unsuccessful.

In summary, NADC30-like strains have become the absolutely dominant epidemic strains in Xinjiang, China. The differences in the number and locations of amino acid mutation sites in the GP5 protein of various PRRSV strains underscore the significant genetic diversity and evolutionary complexity of the virus. Although overall herd immunity levels are relatively high, the protective efficacy of existing vaccines against circulating strains remains insufficient. The commercial vaccine strains currently in use (e.g., CH-1a, VR2332, R98, JXA1-R, TJM-F92, GDr180) mostly belong to Lineage 5 and Lineage 8, which are genetically distant from the currently prevalent NADC30-like strains (belonging to Lineage 1), resulting in limited immune protection efficacy [41]. Therefore, PRRS control should rely on integrated strategies, including establishing a robust biosecurity system, implementing herd health management, developing safe and effective vaccines, and strengthening routine surveillance. This study provides important data support for the scientific prevention and control of PRRSV in Xinjiang.

## 5. Conclusions

Surveillance of large-scale pig farms in southern Xinjiang, China, from 2023 to 2025 revealed that the dominant PRRSV strain in the region is NADC30-like, with detected cases of PRRSV-1 infection (including two single infections and one co-infection), indicating the introduction of this subtype and its potential risk of recombination with PRRSV-2. Virulence-associated mutations such as Q13R and K151R were identified in the viral strains, while variations in the GP5 protein neutralizing epitope and vaccine marker site (A137) suggest immune evasion capability. Whole-genome sequencing confirmed a recombination event between an NADC30-like strain and the VR-2332 vaccine strain. It is recommended to enhance surveillance for recombinant strains and PRRSV-1, as well as to develop new vaccines based on local strains. The protective efficacy of current vaccines may be insufficient, and although herd immunity levels are relatively high, it remains necessary to optimize strain isolation techniques and expand sampling to fully understand the epidemiological patterns.

## Figures and Tables

**Table 1:** Primers used in this study.

Target	Primer Sequence (5′ → 3′)	Size (bp)
PRRSV-1-ORF5	F:TGAGGTGGGCTACAACCATT R:AGGCTAGCACGAGCTTTTGT	702
PRRSV-2-ORF5	F:ACCTGAGACCATGAGGTGGGCAA R:CAAACGGCATCTGGAGGTGATGAAT	963

**Table 2:** Information on PRRSV reference strains.

Reference Strains	Time	Country/Province	GenBank Accession Number	Reference Strains	Time	Country/Province	GenBank Accession Number
Lelystad virus	1993	Netherlands	M96262	CH-1R	2008	Heilongjiang, China	EU807840
EuroPRRSV	2003	USA	AY366525	LNWK130	2017	Heilongjiang, China	MG913987
RespPRRS MLV	1998	USA	AF066183	JL580	2014	Heilongjiang, China	KR706343
ATCC VR-2332	1992	USA	U87392	HENAN-XINX	2013	Henan, China	KF611905
NADC30	2008	USA	JN654459	HNjz15	2015	Henan, China	KT945017
IA/2014/NADC34	2014	USA	MF326985	QYYZ	2011	Guangdong, China	JQ308798
BJ-4	1996	Beijing, China	AF331831	GM2	2011	Guangdong, China	JN662424
NMEU09-1	2009	Beijing, China	GU047345	JS2020	2020	Jiangsu, China	MZ342900
TJ	2008	Tianjin, China	EU860248	ZDXYL 2018-2	2018	Jilin, China	MK453050
HB-2(sh)/2002	2002	Hebei, China	AY262352	LNEU12	2014	Liaoning, China	KM196101
Em2007	2007	Hubei, China	EU262603	CN/FJFQ-1/2023	2023	Fujian, China	OR260421
WUH1	2006	Hubei, China	EU187484	XJ/WJQ	2021	Xinjiang, China	OP866758
WUH3	2008	Hubei, China	HM853673	XJ1904-39	2019	Xinjiang, China	MN119309
HUN4	2007	Hunan, China	EF635006	XJ-Z5	2022	Xinjiang, China	PQ835040
JXA1	2006	Jiangxi, China	EF112445	XJ-1	2021	Xinjiang, China	OQ817853
CH-1a	1996	Heilongjiang, China	AY032626				

## Data Availability

In this study, the PRRSV ORF5 gene sequence (XJLETU5-1-38) and the generated whole genome sequence (XJLETUQ2025-7) have been stored in the GenBank public database. The accession numbers for the 38 ORF5 gene sequences are PX123067-PX123104, and the accession number for the whole genome sequence is PX123105.
